# Carotid Intima-Media Thickness in Indian Patients With Non-alcoholic Fatty Liver Disease: A Systematic Review and a Meta‐Analysis

**DOI:** 10.7759/cureus.68439

**Published:** 2024-09-02

**Authors:** Ramesh Kumar, Ruchika Narayan

**Affiliations:** 1 Gastroenterology, All India Institute of Medical Sciences, Patna, IND; 2 Radiodiagnosis, All India Institute of Medical Sciences, Patna, IND

**Keywords:** from india, atherosclerotic cardiovascular disease, carotid atherosclerosis, carotid intima-media thickness (cimt), non-alcoholic fatty liver

## Abstract

There is a significant association of non-alcoholic fatty liver disease (NAFLD) with cardiovascular disease (CVD). Most CVDs begin with atherosclerosis in the arteries, which can be reliably measured as the carotid intima-media thickness (CIMT) by ultrasound. Given that ethnic and regional differences have an impact on NAFLD, we aimed to evaluate the association of NAFLD patients from India with subclinical atherosclerosis, measured as CIMT.

A thorough literature search was performed on four electronic databases using combinations of several keywords. The relevant data were pooled in a random or fixed-effect model, based on heterogeneity, to calculate the pooled standardised mean difference (SMD), or odds ratio (OR) with 95% confidence interval (CI).

The final analysis included a total of 15 studies with 1196 NAFLD and 1482 control subjects. NAFLD patients had a 21.3% higher mean CIMT than the controls. The pooled SMD was 1.001 (95% CI: 0.627-1.375, p < 0.001). Three studies that balanced cardiometabolic risk factors found a similar association (p = 0.037). Furthermore, NAFLD was significantly associated with the presence of high (>0.8 mm) CIMT (pooled OR = 5.4, 95% CI: 2.0-14 .9) and carotid plaques (pooled OR = 10.24, 95% CI: 5.74-18.26). The mean CIMT was also higher in diabetic NAFLD than in the diabetic control (pooled SMD = 1.07, 95% CI = 0.818-1.324, p < 0.001).

There is a significant positive association between the marker of subclinical atherosclerosis and NAFLD in India. This might give more light on screening and follow-up plans for such patients.

## Introduction and background

Non-alcoholic fatty liver disease (NAFLD) is becoming the most common cause of chronic liver disease due to its rising incidence rate worldwide [1-3]. It is no longer thought to be a condition exclusive to Western nations; rather, the incidence rates are steadily rising across Asia [4]. The estimated pooled prevalence of NAFLD in the adult Indian population is 38.6% (95% CI: 32-45.5%), which is higher than the global average prevalence rate of 30.2% (95% CI: 28.7-31.7%) according to an updated meta-analysis of 479 studies from 38 countries [3,4]. Given its strong association with diabetes mellitus (DM), obesity, and hyperlipidaemia, NAFLD is regarded as a hepatic manifestation of metabolic syndrome [5].

The link between NAFLD and cardiovascular disease (CVD) has been known for long. In fact, CVD rather than liver disease is the main cause of death for these patients. Several studies have demonstrated that NAFLD is an independent risk factor for CVD [5-8]. Therefore, it is crucial to identify NAFLD patients who are at an elevated risk of CVD. Most CVDs start with atherosclerosis in the arteries, which often begins at an early age and progresses over time. NAFLD has also been found to be a risk factor for early atherosclerosis, implying that the association also extends to subclinical stages of CVD [9]. This can have significant ramifications for the early detection and assessment of CVD in patients with NAFLD. By detecting NAFLD at an early stage of atherosclerosis, we may be able to take preventative measures. Carotid intima-media thickness (CIMT) measurement by ultrasound is a real-time safe and reliable tool for the evaluation of the early stage of atherosclerosis. There is a significant association between an increased CIMT and the occurrence of CVD events [1,9,10].

A large number of research had evaluated the association between NAFLD and CIMT [9,11-14]. While the majority of earlier studies were from the Western world, in India, such association has been studied mainly during the last decades. Because NAFLD is influenced by ethnic and regional differences, the CVD risk and the association with CIMT may also vary geographically [15,16]. NAFLD is observed to develop in Asian people in younger populations and those with lower body mass index (BMI) [17,18]. NAFLD in lean subjects has frequently been reported from India [18,19]. There are currently very few studies from India in the published meta-analysis on the association between NAFLD and CIMT [9]. Therefore, we aimed to evaluate the relationship between NAFLD and subclinical atherosclerosis, measured as CIMT, in patients from India.

## Review

Materials and methods

This study was conducted and reported according to the Preferred Reporting Items for Systematic Reviews and Meta-analyses (PRISMA) statement.

Search Strategies

A thorough literature search limited to the English language was performed on electronic databases through PubMed, Google Scholar, Ovid Medline, and Cochrane Library, up until June 2024. The reference lists of the retrieved articles were also examined. The search was conducted using medical subject headings terms and a combination of the following keywords: (Fatty liver disease OR non-alcoholic fatty liver disease OR NAFLD OR hepatic steatosis) AND ((atherosclerosis OR subclinical atherosclerosis) OR (“Carotid Intima-media thickness” OR CIMT OR carotid doppler OR “‘carotid plaque”) AND (“India” OR “Indian”).

Inclusion Criteria and Quality Assessment

The inclusion criteria were original studies investigating the association of adult Indian NAFLD patients (over 18 years) with CIMT. The eligibility criteria were based on the Patient Intervention Comparator Outcome (PICO) framework as follows: (1) P: adult patients with NAFLD diagnosed on any imaging modality; (2) I: carotid Doppler USG examination; (3) C: non-NAFLD control subjects; and (4) CIMT measurement. The quality of observational studies was evaluated using the Newcastle-Ottawa Scale (NOS). Three domains are used in this system to assess the quality of a study: comparability, outcome or exposure, and selection. The study received ratings of poor (0-3 stars), medium (4-6 stars), or high (≥7 stars) based on its quality.

Data Extraction and Analysis

The full text of each article was evaluated and all the relevant data were extracted on a data sheet. The statistical analysis was carried out using the R-based JAMOVI 2.3.28 statistical tool, and the results were shown using forest plots. The standardised mean difference (SMD) with a 95% confidence interval (CI) was computed for continuous data, while log odds ratio (OR) with 95% CI was utilised for dichotomous data. The frequency data from individual studies were summarised as proportions with 95% CI. The threshold for a significant difference was set at p <0.05. Subgroup analyses were performed on the diabetic population and on the basis of confounder adjustment, plaque frequency, and high CIMT prevalence rates. Measures of heterogeneity and publication bias were identified using the I^2^ statistic and funnel plots, respectively. The random-effects model was applied in case of high heterogeneity (I^2 ^> 50%); otherwise, the fixed-effects model was taken into consideration. Sensitivity analysis was carried out following the exclusion of studies with a high risk of bias.

Result

**Figure 1:**
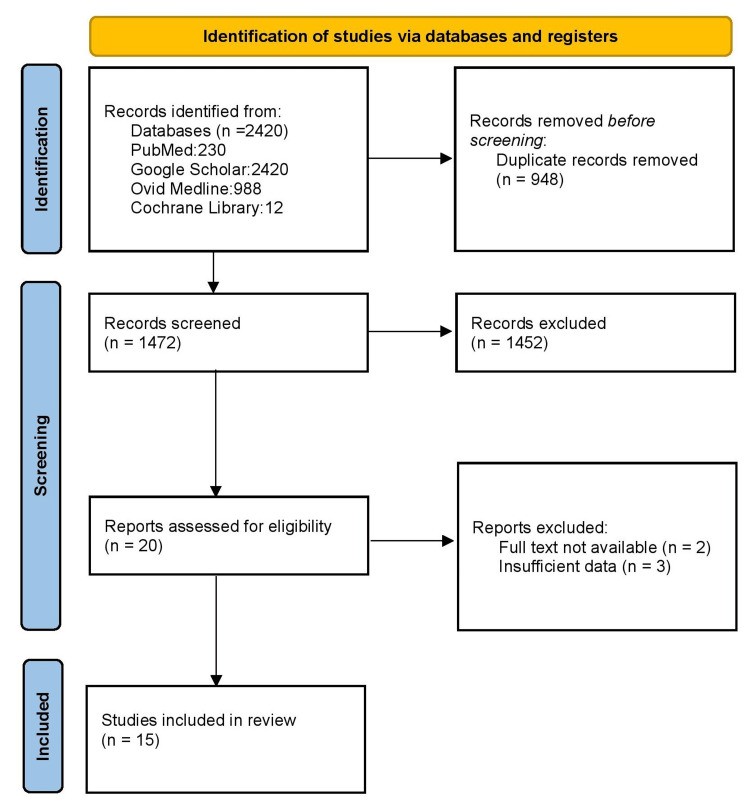
Flow chart showing the search strategy and selection of studies.

Following the initial search that yielded 2420 publications, 2029 articles were excluded only on the basis of duplication or abstract and title review. A total of 20 titles were subjected to a thorough evaluation; of those, five were further removed (Figure 1). Finally, the meta-analysis comprised a total of 15 studies, including 1196 NAFLD patients and 1482 controls.

Table 1 depicts the characteristics of all the included studies. All studies were cross-sectional in design. The mean BMI of NAFLD patients in most studies varied from 22.3 to 28.9 kg/m^2^. On quality parameters, 10 studies (66.7%) were categorised as medium quality, while four and one study were categorised as high and low quality, respectively (Table 1). The mean differences in CIMT between NAFLD and controls were documented in 15 studies overall. Among them, three studies [14,20,21] also provided the mean differences following the adjustment for age, gender, and cardio-metabolic confounders.

**Table 1:** Details of studies included in the meta-analysis CIMT: carotid-intima media thickness; SD: standard deviation; NAFLD: non-alcoholic fatty liver disease; NOS: Newcastle Ottawa scale.

Study	Year	Place	Total N	NAFLD N	CIMT (mean ± SD)		NOS (stars)
					NAFLD	Control	
Agarwal et al. [11]	2011	Delhi	124	71	0.71 ± 0.19	0.67 ± 0.22	6
Thakur et al. [12]	2012	Delhi	80	40	0.6 0 ± 0.12	0.48 ± 0.10	7
Mishra et al. [13]	2013	Jaipur	645	101	0.59 ± 0.10	0.48 ± 0.13	7
Narayan and Ahmad [14]	2024	Patna	150	84	0.75 ± 0.21	0.62 ± 0.23	6
Rasool et al. [20]	2017	Kashmir	300	200	0.84 ± 0.14	0.57 ± 0.10	6
Chouhan. et al. [21]	2017	Gwalior	168	46	0.72 ± 0.17	0.65 ± 0.10	5
Verma and Gour [22]	2016	Bhopal	200	100	0.81 ± 0.10	0.59 ± 0.10	3
Guleria et al. [23]	2013	Chandigarh	40	20	0.7 ± 0.11	0.61 ± 0.01	7
Narayan et al. [24]	2020	Bhubaneshwar	157	58	0.73 ± 0.00	0.66 ± 0.01	7
Vanjiappan et al. [25]	2018	Puducherry	124	73	0.82 ± 0.20	0.64 ± 0.14	6
Hussain et al. [26]	2022	Varanasi	100	76	0.71 ± 0.15	0.58 ± 0.11	6
Rampally et al. [27]	2020	Bengaluru	90	45	0.65 ± 0.20	0.53 ± 0.10	6
Basavaraju et al. [28]	2017	Mysuru	200	62	0.69 ± 0.14	0.54 ± 0.13	6
Raman et al. [29]	2020	Tamil Nadu	200	100	0.85 ± 0.27	0.69 ± 0.16	5
Bajaj and Prajapati [30]	2022	Allahabad	210	120	0.93 ± 0.34	0.81 ± 0.36	5

Association Between NAFLD and CIMT

**Figure 2:**
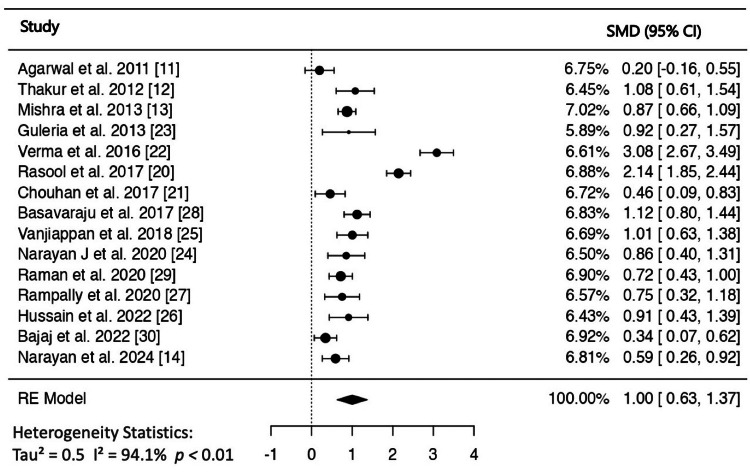
Forest plot of the association of non-alcoholic fatty liver disease with carotid intima-media thickness. SMD: standardised mean difference; RE: random effect.

**Figure 3:**
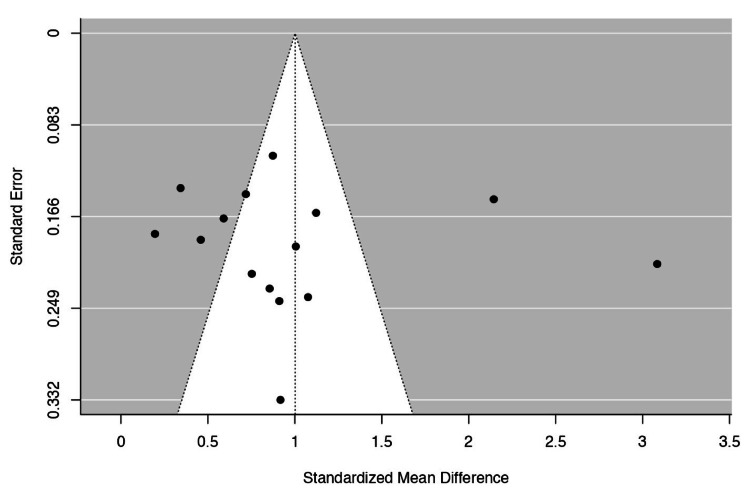
Funnel plot of included studies evaluating association between non-alcoholic fatty liver disease with carotid intima-media thickness. Each plotted point represents the standardised mean difference and standard error for a single study. The plot shows overall symmetry despite a few outliers. The studies are evenly distributed around average effect size. Furthermore, neither the rank correlation (p = 0.32) nor the regression test (p = 0.78) showed any funnel plot asymmetry, indicating the absence of significant publication bias.

The mean CIMT was 21.3% (0.13 mm) higher in the NAFLD patients than in the control group. When data from 15 studies were pooled in the random-effects model, the estimated pooled SMD was 1.001 (95% CI: 0.62-1.37, p < 0.001) (Figure 2). The heterogeneity among the studies was high (I^2 ^= 94%); however, there was no evidence of publication bias. Neither the rank correlation nor the regression test indicated any funnel plot asymmetry (p = 0.3282 and p = 0.7821, respectively) (Figure 3). According to Cook's distances, two studies were deemed to be overly influential [20,22]. Heterogeneity decreased from 94% to 70% when these two studies were eliminated, with an SMD of 0.77 (95% CI = 0.59-0.95, p < 0.001). Furthermore, the pooled SMD did not change after additional sensitivity analysis of the remaining studies, which was carried out by eliminating each study one at a time to determine its impact on the overall meta-analysis estimate. 

In three studies [14,20,21] where CIMT values were provided after adjusting the cardio-metabolic confounders, the pooled SMD was 1.09 (95% CI = 0.06-2.10, p = 0.037, I^2^ = 95%) without publication bias, hinting towards a possible independent association of CIMT with NAFLD. In two studies [23,24], controls included patients with chronic viral hepatitis, while in three other studies [11,25,26], controls were exclusively diabetic patients. To assess the difference in CIMT between NAFLD and healthy controls, we did a meta-analysis after excluding these five studies. The pooled SMD was 0.75 (95% CI = 0.54-0.97, p < 0.001, I^2^ = 72%), suggesting NAFLD patients had higher mean CIMT than healthy controls. In studies where both NAFLD and controls were diabetic [11,25,26], the mean CIMT was again higher in NAFLD subjects than in controls, with the pooled SMD of 1.07 (95% CI = 0.81-1.32, p < 0.001, I^2^ = 0%), with no publication bias. Few studies [14,20,21] also demonstrated a stepwise increase in CIMT with increasing grades of fatty liver on ultrasound.

Association of NAFLD With High CIMT

**Figure 4:**
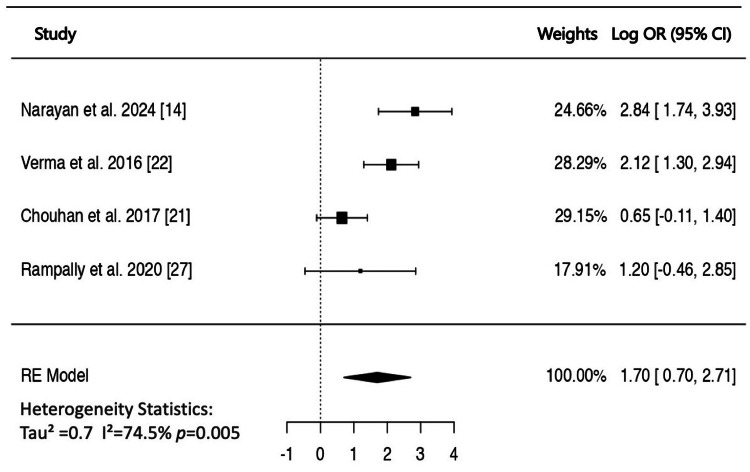
Forest plot of the association of non-alcoholic fatty liver disease with high carotid intima-media thickness (>0.8 mm) OR: odds ratio; CI: confidence interval; RE: random effect.

Five studies have compared the proportion of patients with high CIMT between NAFLD and controls [12,14,21,22,27]. The high CIMT was defined as >0.8 mm in three studies, and >0.9 mm in one study. One study [12] was excluded from the meta-analysis because the cut-off point for high CIMT was unusually low (>0.58). When data from four studies were pooled in a random-effects model, the estimated pooled log OR was 1.70 (95% CI: 0.69-2.70, p < 0.001, I^2^ = 74%), indicating a significant association between NAFLD and high CIMT (Figure 4). The corresponding pooled OR was 5.4 (95% CI = 2.0-14.9).

Association of NAFLD With Carotid Plaque

**Figure 5:**
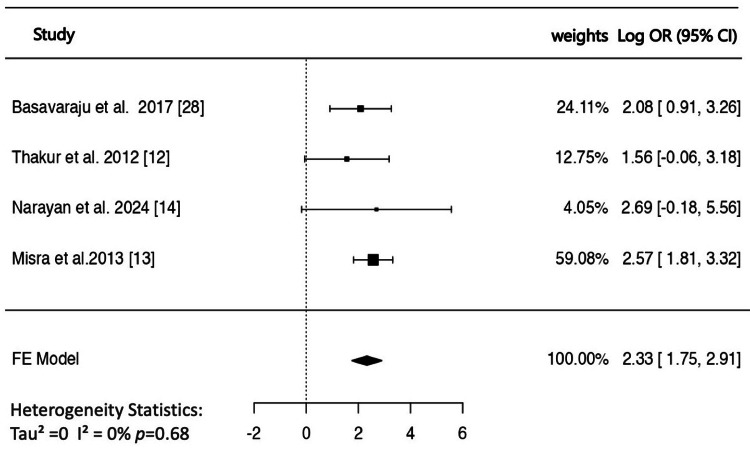
Forest plot of the association of non-alcoholic fatty liver disease with carotid plaques. OR: odds ratio; CI: confidence interval; FE: fixed effect.

A total of four studies [12-14,28] that reported atherosclerotic plaque prevalence were included in the analysis. The estimated pooled log OR based on the fixed-effects model was 2.32 (95% CI: 1.74-2.90), suggesting a significantly greater prevalence in NAFLD patients than in the controls (Figure 5). The corresponding pooled OR was 10.24 (95% CI: 5.74-18.26). There was no significant heterogeneity in the true outcomes (Q = 1.48, p = 0.68, I² = 0%), and there was no evidence of funnel plot asymmetry in either the rank correlation or the regression test (p = 0.75 and p = 0.58, respectively). In one study, the accuracy of plaque score in predicting intracranial artery stenosis was shown to be higher than that of CIMT [29].

Discussion

In this meta-analysis, we synthesised the results of 15 relevant studies (n = 2788) to assess the correlations of NAFLD in Indian patients with subclinical atherosclerosis, as determined by CIMT [11-14,20-30]. We have shown that patients with NAFLD are more likely than the controls to have subclinical atherosclerosis. The mean CIMT was significantly higher in NAFLD patients not only than the healthy controls but also the disease controls consisting of patients with chronic viral hepatitis. These results might shed more light on screening and follow-up plans for NAFLD patients in India. Overall, our data are consistent with the body of global research, indicating that NAFLD is a significant atherogenic risk factor in both Indian and Western populations [2,9].

The significant association between NAFLD and CIMT, even after adjusting for common cardiometabolic risk variables, suggests that it is more than just an epiphenomenon, and NAFLD may have a direct role in the pathophysiology of atherosclerosis. There is mounting evidence that NAFLD stands alone as a risk factor for CVD. NAFLD actively contributes to insulin resistance, oxidative stress, and the production of proatherogenic mediators, all of which increase the risk of atherogenesis [7,8]. The levels of several potential mediators of atherosclerosis, including interleukin-6, oxidised low-density lipoprotein cholesterol, and tumour necrosis factor-alpha, are elevated in NAFLD patients [31,32]. Patients with non-alcoholic steatohepatitis have more atherosclerosis than those with simple steatosis, which further supports the atherogenic effect of hepatic inflammation [33]. Since NAFLD was mostly assessed by ultrasound, the relationship between NAFLD severity and CIMT could not be assessed in our meta-analysis. Some studies [14,20,21] have demonstrated a stepwise increase in CIMT with increasing grades of fatty liver on ultrasound; however, we know that there is a poor correlation between ultrasonographic gradings and the histological severity of NAFLD. 

One of the major risk factors for atherosclerosis and CVD in humans is DM. There are conflicting reports about the relationship between NAFLD and CIMT in the diabetic population [34-36]. While some studies revealed no significant connection [35,36], others indicated a significantly elevated CIMT in diabetic NAFLD as compared to the non-NAFLD diabetic controls [34]. These discrepancies could be attributed to variations in the methodology, such as the diagnostic techniques, the duration of DM, the confounding effects of the medication, ethnic disparities, and so on. According to our subgroup analyses of three studies, the association between NAFLD and CIMT was significant among the diabetic population as well, suggesting NAFLD may have an additional atherogenic effect in patients with DM.

In comparison with the controls, a larger proportion of patients with NAFLD had increased CIMT levels. Nonetheless, the cut-off for identifying an increased CIMT varied between the studies. Generally, a CIMT cut-off at the 75th percentile in a given normal population is considered significant. This cut-off equated to 0.58 mm to 0.60 mm in Indian studies [12,14]. However, a further higher greater cut-off is better correlated with unfavourable results. In the Western population, CIMT >0.90 mm correlates better with organ damage, while the corresponding cut-off for the Indian population is predicted to be 0.8 mm [37,38]. Indians may therefore be more susceptible to CVD at lower CIMT cut-offs. Studies conducted in India revealed that over half of NAFLD patients had CIMT cut-offs >0.80 mm, indicating a high risk of CVD [14,21,22]. Two studies estimated the projected cardiovascular event at 10 years by calculating Prospective Cardiovascular Munster Study (PROCAM) scores and found a significantly higher score in NAFLD patients compared to the controls [23,24]. However, none of the studies followed up the patients prospectively to see actual CVD events in NAFLD patients with respect to the CIMT values. The proportion of patients with atherosclerotic plaque in NAFLD was considerably greater than in controls. Notably, atherosclerotic plaques in the carotid arteries are a better indicator of the risk of CVD and ischaemic stroke than CIMT [39,40].

Although this is the first comprehensive meta-analysis of published studies on the association between Indian NAFLD and CIMT, all of the studies were observational in design, leaving a chance of unmeasured confounders. Moreover, an observational study cannot be used to infer a causal relationship. Heterogeneity was constantly evident in the various outcomes. Furthermore, since all studies used ultrasonography to diagnose NAFLD, misclassification errors cannot be completely ruled out because ultrasonography has a low sensitivity for mild hepatic steatosis.

## Conclusions

This meta-analysis concludes a significant association of NAFLD patients in India with CIMT, which is a sensitive marker of subclinical atherosclerosis. The mean CIMT of NAFLD patients was 21.3% (0.13 mm) greater than that of the control group. The association between CIMT and NAFLD was also present in the diabetic subjects. Moreover, we noted a significant association between NAFLD and atherosclerotic plaques in carotid vessels. The correlations with CIMT are comparable to those found in the Western world, even though Indian NAFLD patients have lower levels of adiposity. These results may shed additional light on the screening and follow-up programs for NAFLD patients in India, as well as the creation of preventative and early intervention strategies for CVD in NAFLD patients. Nevertheless, since our findings are based on cross-sectional research, additional high-quality cohort studies and randomised controlled trials are required to ascertain the clinical importance of elevated CIMT in NAFLD patients and to draw more robust conclusions.
